# Amalgam for Filling Teeth

**Published:** 1856-04

**Authors:** 


### AMALGAM FOR FILLING TEETH.

We give our readers the following discussion on the use of amalgam for filling teeth—that they may get the views of some of our eastern brethren on the subject. We are not prepared as yet to say that the new mode of preparing the amalgam will in all cases resist oxydation—we think not, where the secretions of the mouth are in an unhealthy state.

We have occasionally, for several years past, made amalgam plugs, with pure precipitated silver and tin foil—using merely in gross about two parts silver to one of tin. After mixing these with the mercury, we have been in the habit of washing the mass thoroughly, sometimes with chloric ether and sometimes spirits of camphor or brandy, using whichever happened to be at hand. Such fillings, in healthy mouths, do not discolor. These fillings have almost always been put in when the teeth had been injured by clasps or where they were only expected to subserve a temporary use until the patient might be prepared for a more general operation. We admit that such teeth have often lasted so well and proved so much better than we expected that we have often felt tempted to use it in other bad cases in the molar teeth. Yet when we have come to look farther and examine the effects on other fillings in the mouth, we have and still do hesitate. We are averse to putting in two or three kinds of metallic fillings in the same mouth.

We have, however, felt on this subject a little as we feel in relation to the use of calomel in general practice. The *case* must determine its use. It is my place and duty, as a Physician, to do the best for my patient, and I should be the judge. We *proscribe* nothing and think that calomel if judiciously used is just as *health-giving* and effective for the cure of disease as strychnine, arsenic, opium, emetic tartar, camphor, sage tea or sugar. If we can satisfy ourself that an amalgam plug would be the best for a given case and best for our patient, we should use it.

We know there are many cases where such would not be the case. There are many cases where we have our doubts, and we think that the best way to decide the doubtful cases is to put in gold. The balance of the cases, perhaps, had better be treated as the Doctors advise cucumbers—salt and pepper them well and then throw them *out*—we might, however, substitute the word *draw* for throw.

There are, however, some questions in connection with this subject which lay deeper than the *apparent* benefit derived from an amalgam plug—for this benefit may be only deceptive. If the general health of the mouth, if other filled teeth are really injured by it, the benefit is only deceptive, while, indeed, it may be on the whole very injurious.

If the article is really good, often much better than gold and so much easier used, why not use it in all cases?

If it will not discolor, why not plug the front teeth with it?

The best that can be said of it, however, is, that it is a mere substitute to be used when gold is too expensive or the shape of the cavity does not favor its retention.

We admit that the purity of the metal and the thorough washing makes the article more *tolerable* than as generally used, and we fear that some of the Profession will use it as many of the old Doctors do calomel, in *their* estimation, the *case* always requires it.

The annual stated meeting of the Pennsylvania Association of Dental Surgeons, was held at the Dental College, October 2, 1855, the President, Dr. Daniel Neall, in the chair. The following officers were elected for the ensuing year: President, Dr. Elisha Townsend; Vice-President, Dr. W. W. Fouche; Recording Secretary, James E. Garretson; Treasurer, Dr. David Roberts; Librarian, Dr. S. Dillingham; Committee on Membership, Drs. Edward Townsend, J. H. McQuillen and James E. Garretson.

At this, and an adjourned stated meeting, held October 9, 1855, the attention of the members present was engrossed by the necessary business of the Association. Reports of committees, election of officers, election of new members, (the following gentlemen having been duly elected active members: Dr. Daniel McFarland, Washington, D. C., Dr. John

H. Githens, of Philadelphia, and Dr. C. A. Kingsbury, of Mount Holly, N. J.) the appointment of a committee to superintend the publication of the transactions of the society, (the columns of the Dental News Letter having been offered to the association for that purpose by Dr. J. R. McCurdy,) the adoption of an amendment to the by-laws; changing the Examining Committee to a Committee on Membership; the appointment of a committee for the purpose of increasing the society's library, and lastly, Prof. White was requested, by the President, to prepare a dissertation for the next stated meeting.

A special meeting of the Association was held at the Dental College, October 16, 1855, the President, Dr. Elisha Townsend, in the chair. Members present, Drs. Arthur, Williams, Neall, White, Pierce, Buckingham, Dillingham, Calvert, Flagg, Harris, Roberts, McCurdy, Du Bouchet, Edw. Townsend, Garretson and McQuillen.

The principal interest of the meeting was the consideration of the amalgam question, as presented in the Dental News Letter by Dr. Elisha Townsend. The doctor, at the desire of the meeting, arose and said: He was glad to have the opportunity of defining his position in this matter; felt and knew that he had been misunderstood; wished that his views should be made very clear, and the sincerity of his sentiments appreciated; alluded to the length of time he had been in practice; to the strides toward perfection the profession had made in that period; the exposition of old ideas and prejudices, and the adoption of new and true ones; experience proving their truth. Few bodies of men were ever joined together for a common good, who had individually labored more honestly, or with more singleness of heart for the good of the whole.

He was proud of his profession and of his brethren engaged with him in its cultivation, and was unwilling the antiquarian spirit should bury him in the relics of the past. If, in what I now have to say to you, you deem me in error, do not hastily condemn the suggestion I make, but let me ask you, each and all, to experiment in the article of amalgam, with an eye single and sincerely desirous of eliciting the truth, whatever that may be—be careful in your experiments and in all your manipulations; do not take anything upon trust from me, or use it blindly and indiscriminately because I do, but see for yourselves. "Mark, learn and inwardly digest," before you add your weight of sanction to mine in giving character that after all may not belong of right to the article of which I speak. By thus doing you will strengthen and aid me if I am right, and will deserve the thanks of the profession and the public as well as mine. If I am wrong, and you can convince me of it, you shall have my sincere and heartfelt thanks. I wish the greatest good of the profession, and that through its increasing knowledge, its usefulness may be so increased that it shall be an honor and privilege to be ranked among its cultivators. You are aware, from my article in the News Letter, that I have occasionally used the article of amalgam in my practice, and I think my duty to my patients compels me to do so. The new method of preparation rids it of the greatest objection to it—its blackness, and scientific experiment has proved that it cannot be injurious to the mouth or teeth in any other way. The present mode of preparation, we think, secures it from the possibility of discoloration. Do not misunderstand me ; I do not advocate the use of it to the exclusion of gold, or in cases where a solid filling of gold can be placed, but there are teeth worth saving, even if for a short period, where any filling requiring pressure to compact it would be inadmissable, and here a plastic material is invaluable. I know there are some prodigies who *never* are baffled, who *never* see a tooth they cannot fill with gold. I am *not* a prodigy, and I do often see teeth my patient will thank me for saving, if even for a few months, which I have not the skill to fill with *gold.*

The Doctor here presented an artistical crown of amalgam built upon the fangs of an inferior molar. This, gentlemen, is the counterpart of an operation performed in the mouth of a lady, a few weeks since ; she came to me to have it extracted, and is now using it with perfect comfort with its artificial crown. The manner of building it was thus : a piece of watch spring well annealed was carefully fitted around the fang, passing under the free margin of the gum ; into this the filling was packed and built up to the hight necessary for the proper antagonization with the superior tooth ; the spring was allowed to remain for an hour, then removed and the edges trimmed and packed and burnished. In two days the patient returned, when the filling was stoned and polished, as the one I present to you. After some remarks on the chemical affinity of the metals used, the Doctor said that as long as there was no loss of substance, no disintegration of the particles of the filling, there could be no systematic effect produced.

A member asked, "if the metals employed were required to be pure?"

Certainly, the mercury of the shops has to be purified chemically.

[By request, Dr. T. here prepared some amalgam, according to the recipe given in the News Letter, and filled a tooth in presence of the members.]

Dr. Jas. M. Harris expressed admiration for the article, and hoped it might not disappoint Dr. Townsend's most sanguine expectations, as he thought it promised to be a great aid to dentists; spoke of many teeth sacrificed after being filled with gold or tin, in consequence of inflammation ensuing, which might have been saved, if no pressure had been used in packing the filling; thought the matter of great consequence, and hoped no prejudice might be allowed to influence the decision which should follow a close examination of its merits.

Dr. Townsend—Let me be understood by my friends to be only an experimenter, and perhaps I have only given you one bright side of this subject; there may be a blackened side, oxydized like the old succedaneum; I have not yet seen it; when I do, I shall tell you. But while I can put a ring around a tooth which has been cut or worn by a spring or band, and after a few hours replace the band which can be worn comfortably without being affected by the mercury; while I can build up large crowns of teeth on mere fangs, useful for mastication, and find no injurious systematic or local effects, I feel bound in honor and duty to my patients to employ it. Dr. Harris' manner of receiving the subject pleases me. I want my professional brethren to work with me, to assist in building up or tearing down. Don't take hold of it, pinning your faith on my sleeve. If it is a good thing, let us test it and convince ourselves; if it is not, the sooner we know it the better. If it be a truth, it will stand and need no backers—if not, it will as certainly fall. Let me express the hope that you entirely understand my sentiments and connection with this question. I do not, as yet, endorse the use of amalgam, except as an experiment; but time, which proves all things, will prove this.

Prof. Buckingham alluded to numerous experiments he had made with amalgam, which, in the aggregate, had resulted unsatisfactorily; spoke of the action of acids on metals as influenced by proportions of alloy; considered the character of the new amalgam to be greatly influenced by the length of time the superfluous mercury was allowed to remain in conjunction; exhibited specimens, one of which had been amalgamated over night, and the mercury pressed from it on the ensuing morning; the material presented a soft and pasty character. Another specimen, prepared just before filling the cavity, looked firm and silvery; thought, however, (and offered the suggestion as the result of continued observation,) that teeth which were so far decayed as to prevent the introduction of gold or tin fillings had better be removed from the mouth, than be allowed to remain in it. In conclusion, remarked that even admitting this new amalgam to possess some virtue, the harm which would result from its general use, would so far counterbalance the good, as to make its endorsement unadvisable.

Dr. Daniel Neall belonged to the progressive party: was unwilling to condemn anything without a fairer trial than the new formula had received; always stood on the threshold to welcome improvements and suggestions; thought that instead of condemning hastily, it might be as well to convince ourselves by experimenting; desired to know whether Dr. Townsend would fill all frail teeth with the amalgam?

Dr. Townsend—By no means; such alone as I deem may be saved, but not with gold foil.

Dr. M'Quillen had no experience to offer in the use of the article, having filled but one tooth with it, and that was out of the mouth. Before experimenting in it, let alone introducing it into his practice, he desired to obtain all the light he could upon the physical and medical properties of mercury; alluded to the views entertained by Fownes, Turner, Faraday and other chemical writers, that mercury volatilizes to a sensible extent at all temperatures above 70°, but does not oxydize at any other temperature than a little below its boiling point, 62°; dwelt upon the difference of opinion between writers. Wood and Bache, regarding metallic mercury as inert in its combination with other elements; admitting, at the same time, the possibility of such combination taking place in the system when retained for any length of time. On the other hand, cited cases from Dunglison's Therapeutics, where individuals had unquestionably been affect ed by metallic mercury; considered the presence of mercury in any preparation intended to be used as a filling for decayed teeth as decidedly objectionable; had reason to believe that in the most perfect amalgam filling, there would be a certain amount of free mercury present, which would undoubtedly volatize, exposed as it must be to a temperature near 98°; alluded to the idiosyncracy many persons labored under where the administration of the slightest amount of mercury is sure to be followed by serious results.

Dr. Githens had filled many teeth with amalgam, but only used it in cases where gold foil could not be employed. Several years since, at the urgent request of a patient, filled a number of teeth with amalgam, which at the time he thought ought to be extracted, and so advised, but overruled, allowed himself, more as an experiment, to use the amalgam, suggesting that in all probability they would last but a few months; saw them five years afterwards, and the teeth looked as well as when the operation was performed. In another case, had preserved certain teeth fifteen years when filled with amalgam fillings; thought that by the use of gold they could not have been preserved as many months; could not understand why in such cases amalgam should be repudiated; never knew of any injury resulting from its use, systemic or local.

Prof. Flagg—When the article was first introduced into the country, many years ago, had, in connection with his brother, violently opposed the use of it; wrote, he believed, the first article penned in opposition in this country, which was published in the Boston Medical Gazette; had changed his views, and was using the new formula in his practice; inclined to the belief that for such cases as recommended it is invaluable. Some time since saw a case of ptyalism supposed to result from the presence of four amalgam fillings; thought, if the deduction was correct, the fault lay rather in the manipulations than in the material, the amalgam having been plastered roughly in the cavities and around the necks of the teeth; otherwise had never seen anything like an authenticated case; thought the stigma attached to the article influenced the opinions of practitioners, which, to say the least, was unscientific; remarked that if an amalgam filling should oxydize, (but which, if properly prepared, it is believed cannot be the case), a little pumice will soon remove the objection.

Prof. White.—The article as yet is but an experiment; a few months experience could tell nothing about it. The objectionable features of the old preparation being present in the new formula, had reason to believe that a few months would convince its present advocates of the impropriety of using it in their practice.

Dr. J. M. Harris related cases coming under his observation where the merest shell of teeth had been preserved twenty years by the aid of amalgam; the only apparent objection being the discoloration; hoped the new mode of preparation would clear up this objection believed it would.

On motion, the discussion was continued to the next special meeting, November 20th.

A special meeting of the Association was held at the Dental College on the evening of November 20th, 1855. The President, Dr. Elisha Townsand, in the chair. Members present: Drs. Daniel Neall, Fouche, Pierce, Harris, Du Bouchet, Calvert, McQuillen, Dillingham, Flagg, Buckingham, McCurdy and Garretson.

The president having stated that the subject for discussion was the amalgam question, as continued from the last meeting, requested the views of Dr. Fouche.

Dr. Fouche had formerly been opposed to the amalgam for filling teeth, and up to a few months back never used it, but in the new formula found his objections removed; has been carefully experimenting, and is convinced that in this preparation we have a desideratum long sought, and recomends it to the profession as an invaluable aid in such cases as it is intended to be used; has filled as many as five teeth in a day with it; so far as the short time admits of a proper judgment, his operations have been attended with the best results.

Dr. Townsand desired the grounds of Dr. Fouche's former objections.

Dr. Fouche supposed that ptyalism was sometimes caused by the use of amalgam for filling teeth, and again its rapid oxydation; all his fillings of the new formula remain as bright and silver-like as when first placed in the mouth, a period of four months; inclined to the belief that the impurity of the metals used, and their imperfect combination, would be a sufficient explanation of the objections to the former material; the present process of trituration, the complete alcoholic washings, the strict enjoinment to use chemically pure metals, removes these objections as he had convinced himself.

Dr. Du Bouchet had used the material in a single case; saw the filling two days after and it had not solidified.

Prof. Flagg had employed it in seventy cases which he thought desperate ; removed only four of the teeth, which were entirely devoid of vitality. Believes, as he stated at the last meeting, that it is a desirable preparation ; but as then recommends it in such cases, as for sundry reasons gold foil may not be employed.

Dr. Dillingham had tried it cautiously ; placed it in many delicate mouths ; watched cases closely, and was most favorably impressed.

Dr. McQuillin had doubts about the correctness of applying the term oxydation to the discoloration that occurs in amalgam fillings. Alluded to the fact that silver and murcury only oxydize at the melting point of one and boiling point of the other ; but admitting at the same time, that new affinities might arise in a state of combination. Would offer as a supposition, (not as an assertion), that the discoloration was due to the affinity that the two metals have for the sulphur contained in the albuminous portions of the food. Cited the blackened appearance of silver spoons when used with boiled eggs ; also the tarnish that forms on silver plate when exposed to the atmosphere ; in the first instance a sulphuret of silver being formed from the sulphur contained in the albumen of the egg, and in the second from sulphureted hydrogen present in the air. Was satisfied that the most careful washing could not overcome the affinities that exist between elementary substances, and beleived that a later experience would prove the possibility of discoloration taking place in the new amalgam.

Dr. Fouche.—If the discoloration that forms in the amalgam is not an oxyde, then he did not profess to know what an oxyde is. Had noticed that the amalgam was not as good a conductor of heat as gold ; and regarded this as an important matter in filling teeth where the nerves are nearly exposed.

Prof. Buckingham could see no particular difference between the so called new and old amalgams, precisely the same metals being used. Pure murcury will not oxydize except at a high temperature, but when amalgamated with other metals it is another matter. This amalgam oxydes in preparing it ; to wash away which Dr. Townsend uses absolute alcohol. What then is to prevent a similar oxyde from forming in the mouth ? Believed it a good thing in its place, but it was evident that its advocates were getting it thus early quite out of its proper position. Thought that in a short time they would discontinue its use.

Dr. Daniel Neall valued the teeth beyond price; as worthy of having invoked to there aid any thing and every thing promising their salvation. Alluded to the teeth which, from peculiar relations, might possibly be saved with something of the kind when they could not be with gold—frail teeth, or peculiar articulating teeth. Never extracted a tooth when there existed a prospect of saving it. Is in the habit of filling the worst teeth with gold, believing it desirable to run the risk of having afterwards to extract, that we may at least enjoy the consciousness of having made the attempt to save: would try every thing before resorting to extraction. Thought this amalgam might prove a valuable addition to our pharmacopia, if kept in its proper place; but to place it on an equality with gold would never meet with his sanction Desired to know whether he understood Dr. Fouche to say that he was in the habit of filling five teeth a day with the amalgam.

Dr. Fouche.—Yes, and large cavities; filling them, as I believe, with greater benefit to my patients than if I had used gold, and with much less fatigue both to them and myself.

Dr. Neall.—There is the difficulty—this wholesale use of the article. In my own practice I do not see five teeth in a week I could not save with gold.

Prof. Flagg.—Surprised at Prof. Buckingham's assertion, "that there was no difference between the new and old formula;" as in the old we had copper, lead, bismuth and other alloys, while in the new we have the pure metals. Remarked that three years had proven the unchanging character of the new formula. Thought that pecuniary considerations should be taken into account. Did not know how it might be in the practice of others, but in his own could often save desirable teeth, where the patient could not afford the expense of gold. Asked if, in such cases, the teeth should be suffered to decay without any attempt being made to save them?

Dr. Pierce.—Would Prof. Flagg prefer the amalgam to tin foil?

Prof Flagg.—Decidedly,

Prof. Buckingham.—As professional men, the question of dollars and cents should never be allowed to stand between us and the patient's best interests. The question should not be presented on such grounds, but only in connection with its merits and demerits. If we consider gold the proper material to be employed, then I think we have no right to use any thing less valuable ; but tin foil had better be used than this amalgam because we have nothing to fear from it, at least in the shape of an oxyde.

Dr. Townsend.—With regard to the prejudices existing against the article, inclined to believe it resulted from amalgam generally being found in company with bad teeth ; teeth that never should have been filled, and again from its being very much employed by quacks ; yet, even in the hands of such persons, has known teeth to be saved for years. Considered that there was sufficient difference between the new formula and the old to remove all objections to its prudent employment. The mass being homogeneous, no lead, or other impurities and no oxyde, as that which forms in trituration, is removed by the alcoholic washings, Dr. McQuillin's views relative to mercury only oxydizing at the boiling point is incorrect, as any one who has prepared bluemass can readily assert. As Prof. Buckingham condemns the amalgam so much on account of the oxyde, would ask if he had never remarked the same result in gold fillings.

Prof. Buckingham.—Often, but the discoloration that occurs under gold fillings and oxydation, are two things.

Dr. Harris had remarked in his practice, one case where ptyalism seemed likely to result from the use of amalgam in filling teeth ; would state the case, that the members might judge whether the fault lay in the materials or in the manipulation; Eighteen teeth had been filled ; the material had been plastered not only in the cavities, but about all the necks, and margin of the gum, making as it were a mass of teeth and amalgam. The cavities seemed to have received no preparatory treatment, and certainly no attempt had been made to finish up a single filling.

Dr. McQuillen.—Notwithstanding all that had been said in favor of the article, and the fact that one operation he had performed in the mouth promised favorably, felt a disinclination to introduce it into his practice. Freely admitted he had never seen a case of ptyalism resulting from the presence of amalgam fillings ; supposed the limited use of the article since his entrance into the profession would account for that. Had, however, every confidence in the judgment and veracity of Prof. Harris, and other writers who assert positively that cases have come under their notice, not only of ptyalism, but extensive absorption of the alveoli, induced by the presence of amalgam fillings. As the active agent that brought about the trouble in these cases was present in the new formula, they could not but regard with misgivings its reintroduction to the profession.

On motion, adjourned.

